# Expression of Cancer-Associated Molecules in Malignant Mesothelioma

**Published:** 2007-05-30

**Authors:** Ben Davidson

**Affiliations:** Department of Pathology, Rikshospitalet-Radiumhospitalet Medical Center, Montebello N-0310 Oslo, Norway

**Keywords:** malignant mesothelioma, hallmarks of cancer, metastasis, high throughput

## Abstract

Malignant mesothelioma (MM) is a malignant tumor derived from mesothelial cells, native cells of the body cavities. Exposure to asbestos is the most strongly established etiologic factor, predominantly for the most common disease form, pleural mesothelioma. The pathogenesis of MM involves the accumulation of extensive cytogenetic changes, as well as cancer-related phenotypic alterations that facilitate tumor cell survival, invasion and metastasis. This review presents current knowledge regarding the biological characteristics of this disease that are linked to the so-called hallmarks of cancer. In addition, data suggesting that the anatomic site (solid tumor vs. effusion) affects the expression of metastasis-associated and regulatory molecules in MM are presented. Finally, recent work in which high-throughput methodology has been applied to MM research is reviewed. The data obtained in the reviewed research may aid in defining new prognostic markers and therapeutic targets for this aggressive disease in the future.

## Introduction

Malignant mesothelioma (MM) is a tumor derived from mesothelial cells, native cells of the body cavities. The pleural cavity is the most common site, with a present ratio of 9:1 with peritoneal tumors. Exposure to asbestos can be documented in about 80% of the cases (Britton, 2002). The incidence of MM appears to be rising steeply in western countries, a trend that is likely to continue (Parker, 2003; van Ruth, 2003). MM is an aggressive and rapidly fatal disease, with a median survival of 8 months if untreated, although selected patients achieve survival of 2 years and more when surgery is combined with adjuvant therapy (Parker, 2003; van Ruth, 2003). Validated clinicopathologic prognostic factors of poor survival are non-epithelioid tumor type, poor performance status, male gender, high white blood cell count and low hemoglobin levels (Burgers, 2004). The disease develops for decades, during which the neoplastic cells accumulate a variety of chromosomal aberrations. Extensive studies using traditional cytogenetics, analysis of microsatellite instability/loss of heterozygosity and fluorescent in situ hybridization (FISH) have shown that gains or losses of entire chromosomes or chromosome fragments most frequently involve chromosomes 1, 3, 6, 7, 9, 14, 18 and 22 (reviewed in Sandberg, 2001; Musti, 2006). Use of gene-specific molecular probes has identified loss of the cyclin-dependent kinase-4 inhibitor (CDKN2A) (Prins, 1998; Illei, 2003), neurofibromatosis type 2 (NF2) (Bianchi, 1995; Sekido, 1995), p15 and p16 (Xio, 1995), p53 (Cote, 1991), and Fhit (Pylkkanen, 2004) genes in MM, supporting the role of cell cycle deregulation and inactivation of tumor suppressor genes in its pathogenesis.

Simian virus 40 (SV40) is an additional postulated contributing factor in the development of MM. Some of the Polio vaccines prepared during the 50’s and early 60’s have been shown to be contaminated with SV40, a polyomavirus that produces an asymptomatic infection in macaque rhesus monkeys, its natural host. Earlier studies have reported high levels of expression of SV40 DNA or large T antigen protein in MM (reviewed in Shah, 2006), although not all studies reproduced this observation (Simsir, 2001; Manfredi, 2005). The presence of SV40 has been associated with expression of several cancer-associated molecules, the majority of which are discussed below. The presence of SV40 in MM was shown to be associated with more frequent methylation of the *RASSF1* gene, a putative tumor suppressor gene in lung cancer (Toyooka, 2001). SV40 replication was associated with autocrine activation via hepatocyte growth factor/scatter factor (HGF/SF) and its receptor Met (Cacciotti, 2001). The presence of SV40 has been shown to be associated with higher levels of vascular endothelial growth factor (VEGF) (Cacciotti, 2002), telomerase activity (Foddis, 2002), expression of the cell cycle inhibitor p21^WAF1/CIP1^ (Baldi, 2002), p53 inactivation (Carbone, 2003) and activation of the phosphatidylinositol 3-kinase/AKT signaling pathway (Cacciotti, 2001; Ramos-Nino, 2005).

The role of SV40 in the pathogenesis of MM has been growingly questioned in recent years. Twenty-one of 31 papers published in the time period from 2002 until the publication of the above-mentioned review have failed to find any evidence supporting the presence of SV40 in MM or found only rare DNA or protein expression of the virus (Shah, 2006). Furthermore, contamination with laboratory plasmids has been shown to be a frequent source of false-positive results in studies analyzing the presence of SV40 (Lopez-Rios, 2004), and serological studies have shown rare seroreactivity for the virus among the general population and MM patients, as well as cross-reactivity with the more prevalent BKV and JCV polyomaviruses (Shah, 2006). These data suggest a need to re-evaluate the role of SV40 in MM.

Patients with the more common pleural MM typically present with cough, dyspnea and chest pain, reflecting the presence of pleural plaques and a characteristically unilateral malignant hemorrhagic effusion (Pass, 1993; Moskal, 1998). Patients with peritoneal disease present with abdominal distention and pain that are caused by the presence of malignant cells in ascites and in solid peritoneal lesions, a clinical picture that may be indistinguishable from that observed in ovarian or peritoneal carcinoma (Eltabbakh, 1999; Sugarbaker, 2003). Although present in a large number of cases at autopsy, clinically detectable distant metastases are rare at presentation.

**Figure 1:**
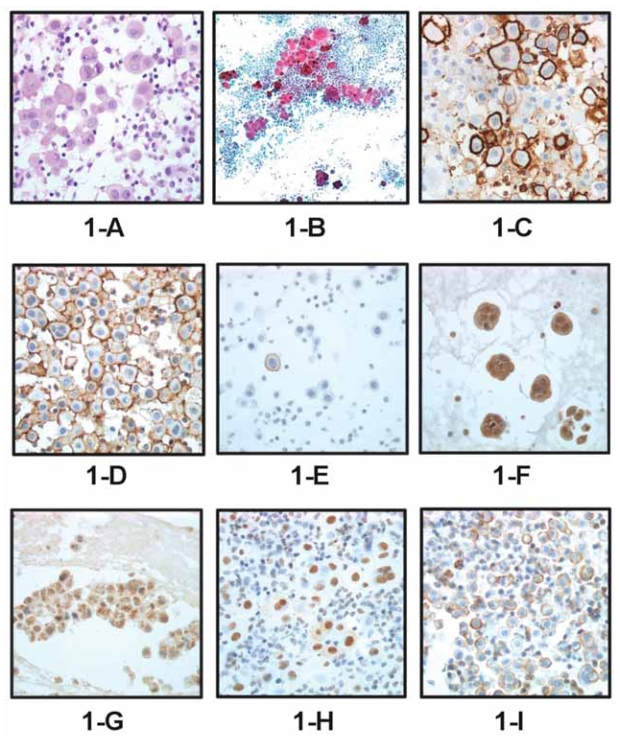
**A.** H&E staining of a cell block section from a pleural mesothelioma; **B:** PAP-stained smear from the same specimen; **C:** Protein expression of the 67kDa laminin receptor, a rare event in mesothelioma; **D:** p-TrkA membrane expression in a pleural mesothelioma; **E:** Focal p75 expression in the same specimen. Only one cell expresses this receptor; **F–G:** Heparanase (F) and bFGF (G) cytoplasmic expression in two pleural mesotheliomas; **H:** Nuclear expression of activated ERK (p-ERK) in the pleural mesothelioma shown in figures A–B; **I:** membrane expression of HLA-G in a pleural mesothelioma effusion.

The main differential diagnosis of epithelioid MM, the most common histologic type, is with metastatic adenocarcinoma, most frequently of ovarian, breast or lung origin, and reactive mesothelium (RM). Benign mesothelial cells react to a wide variety of stimuli and injuries by proliferation and reactive cellular changes that may mimic the morphology of malignant cells (Bedrossian, 1994). These changes may be especially pronounced following radiation or chemotherapy, common adjuncts to surgery in the treatment of various malignancies (Bedrossian, 1994). The overlapping morphology of these cell types does not allow for a reliable diagnosis of MM without the use of ancillary techniques. In recent years, immunohistochemistry has largely taken over the role of electron microscopy in this field. While the possibility to diagnose MM in cytological material is still not universally accepted, improved antibody panels and the use of formalin-fixed paraffin-embedded cell block material for effusion diagnosis have led to results that are at least comparable to those observed in surgical specimens, with lesser morbidity and cost (Figures 1-A, 1-B) (Bedrossian, 1998; Davidson, 2001). In fact, the diagnosis of MM can be reliably obtained within less than one day using flow cytometry, a procedure that is optimal for effusion specimens (Davidson, 2002a; Sigstad, 2005). Recently, molecular methods such as comparative genomic hybridization (CGH) and FISH have been successfully used for the diagnosis of MM in cytological material (Nagel, 2002; Illei, 2003).

Cancer cells show a spectrum of phenotypic aberrations, all reflecting the loss of host regulation and the ability to override checkpoints that allow normal tissue to remain in homeostasis. These consist of altered cellular adhesion, abnormal response to growth-promoting signals, cell cycle deregulation and evasion of apoptosis, and enhanced proteolysis and angiogenesis (Hanahan, 2000). Although the majority of translational correlates that are related to chromosomal changes in MM and the mechanisms of epigenetic regulation in this disease are still undefined, recent research has defined many of the phenotypic characteristics of MM that are related to the so-called hallmarks of cancer. This review presents currently available data regarding the cancer-related biology of MM, with focus mainly on research performed on clinical specimens. Data suggesting that the anatomic site (pleura vs. peritoneum) and growth conditions (solid tumor vs. effusion) may affect the phenotype of MM cells are presented. Finally, recent work in which high-throughput methodology has been applied to MM research is reviewed.

## Proteolytic Enzymes

Matrix metalloproteinases (MMP), a family of zinc-and calcium-dependent enzymes, are central mediators of the biology of tumor invasion and metastasis, due to their ability to degrade the basement membrane and essentially all extracellular matrix (ECM) components (Egeblad, 2002). Two members of the family, MMP-2 and MMP-9, are the proteases involved in basement membrane degradation, a major event in the dissemination of epithelial cancer. MM cell lines have been shown to express and activate MMP-2 and MMP-9, and express and secrete several other members of this family, including MMP-1, MMP-3, MMP-7 and MMP-10, as well as the MMP inhibitors TIMP1–3 (Liu, 2001). MMP expression and secretion by MM cells *in vitro* is increased by several growth factors, including HGF/SF (Harvey, 2000; Liu, 2003), epidermal growth factor (EGF), acidic and basic fibroblast growth factor (aFGF, bFGF), insulin-like growth factors I and II (IGF-I,II) and transforming growth factor-α (TGF- α) (Liu, 2003). MMP-2 and MMP-9 activity, as measured by gelatin zymography, was documented in clinical MM specimens, with predominant activation of the former enzyme. MMP-9 activity was significantly higher than in benign pleura, and MMP-2 was an independent predictor of poor survival (Edwards, 2003). Our group recently found expression of MMP-2, MMP-9, MMP-14 (MT1-MMP) and TIMP-2 and activation of MMP-2 and MMP-9 in MM effusions (Sivertsen, 2006). MMP-2 and TIMP-2 mRNA expression was significantly higher in peritoneal compared to pleural MM effusions (Sivertsen, 2006). The data in the above studies suggest a central role for MMP in the pathogenesis of MM.

## Adhesion Molecules and Other Membrane Receptors

Cell-cell adhesion is essential for normal cellular architecture and homeostasis. Cadherins, a family of Ca^2+^-dependent integral membrane glycoproteins, are located at the cell-cell adherens junctions, where they mediate homophilic contact with neighboring cells (Takeichi, 1991). Early data suggested that MM and AC cells exclusively express the epithelial and neural type of this molecule (E-cadherin and N-cadherin), respectively (Han, 1997). These data have been shown to be erroneous by other investigators and us (Davidson, 2002; Abutaily, 2003; Sivertsen, 2006a; reviewed in Ordonez, 2003a). In fact, MM and serous AC are the two tumors that most strikingly exhibit co-expression of different cadherins, including N-cadherin, P-cadherin and E-cadherin (Sivertsen, 2006a). Furthermore, MM cells in effusions upregulate the expression of these three cadherin molecules, suggesting that they play a role in metastasis and tumor progression (Sivertsen, 2006b). This profile may aid MM cells in combining conserved adhesion between tumor cells with the aggressive behavior of epithelial/epithelioid cells undergoing epithelial-mesenchymal transition (EMT), a process that is characterized by loss of E-cadherin and acquisition of N-cadherin (reviewed in De Wever, 2003). Interestingly, MM cells, including those of the epithelioid type, frequently express neural cell adhesion molecules (NCAM), members of a different family of adhesion molecules that are expressed by neural and neuroendocrine cells and biphasic tumors (e.g. synovial sarcoma) (Lantuejoul, 2000). Seen together with the observation that MM express nerve growth factor receptors (Davidson, 2004), it is evident that this tumor is able to produce a remarkable array of molecules that have been previously thought to be lineage-specific.

Integrins, a second family of adhesion molecules, are heterodimers consisting of noncovalently linked α and β subunits (Hynes, 1992). Integrins are fundamental regulators of cell growth, migration, survival and differentiation, and transduce signals from the extracellular environment to the gene expression machinery, thereby modulating signaling events initiated by growth factor receptors (Howe, 1998; Dedhar, 2000). The extracellular matrix (ECM) integrin ligands include laminin, fibronectin, collagen, vitronectin, entactin, tenascin, and fibrinogen (Ruoslahti, 1991).

Klominek and co-workers previously showed that integrins are expressed in MM cell lines and that they mediate migration towards ECM proteins, including collagen type IV, fibronectin and laminin (Klominek, 1997). Koukoulis et al. studied 22 clinical MM specimens and found that the expression of most integrins, including the α6β4 integrin, in epithelioid MM is similar to previously reported patterns in AC of the lung, and often of other origins (Koukoulis, 1997). We recently found that MM cells frequently express the αv and β1 integrin subunits, and that the α6 subunit, part of the α6β4 and α6β1 laminin receptors, is more frequently expressed in MM compared to ovarian and breast AC (Sigstad, 2005). In contrast, the 67kDa laminin receptor, a non-integrin receptor that is frequently expressed in AC at all sites, is only rarely expressed in MM (Figure 1-C) (Reich, 2005). In view of the recent data of our group regarding the role of laminin receptors in tumor invasion and metastasis via activation of MMP-2 synthesis in malignant melanoma (Givant-Horwitz, 2004), it is conceivable that integrins, rather than the 67kDa receptor, may contribute to MMP production following binding to laminin in MM.

## Angiogenic Molecules and Other Growth Factors and Growth Factor Receptors

MM cells synthesize a large number of angiogenic molecules and other growth factors that may provide autocrine stimulation and relative independence from the stromal and endothelial production of these factors. The most extensively studied molecule in this context is VEGF. Expression of VEGF and its receptors flt-1 and KDR has been shown in several studies, supporting the existence of this autocrine pathway in MM (Konig, 1999; Kumar-Singh, 1999; Ohta, 1999a; Konig, 2000; Strizzi, 2001a; Catalano, 2002; Davidson, 2004). A similar autocrine loop may exist for PDGF and its receptor PDGFR (Ascoli, 1995; Langerak, 1996; Klominek, 1998a). PDGFR is of special interest since the α subunit of the receptor is the predominant one expressed in benign mesothelial cells, whereas expression of the β subunit is found in MM cell lines and clinical specimens (Versnel, 1991; Ramael, 1992). Inhibition of PDGF-β using ribozyme technique results in reduced cell growth concomitantly to reduction in PDGF-β levels in the VAMT-1 cell line (Dorai, 1994).

Additional pro-angiogenic molecules and growth factors that are synthesized by MM cell lines and clinical specimens are interleukin-8 (IL-8) (Galffy, 1999; Davidson, 2004), bFGF (Strizzi, 2001b; Davidson, 2004), TGF-α (Langerak, 1996), IGF (Hoang, 2004a), heparanase (Davidson, 2004), thrombospondin (Ohta, 1999b), HGF/SF (Klominek, 1998b; Thirkettle, 2000) and several members of the Syndecan proteoglycan family (Kumar-Singh, 1998; Gulyas, 2003). MM cells express both EGFR (Dazzi, 1990; Pache, 1998; Manning, 2002; Cai, 2004; Destro, 2006) and its family member erbB-2 (Thirkettle, 2000), and EGFR expression has been shown to be closely related to the pathogenesis of asbestos injury (Pache, 1998; Manning, 2002). However, EGFR mutations were not detected in pleural MM (Destro, 2006).

Microvessel density is an independent prognostic marker in MM (Edwards, 2001). The clinical significance of angiogenic molecule expression is suggested by the observed correlation between VEGF and bFGF and poor survival, with an opposite finding for Syndecan-1 (Kumar-Singh, 1999). EGFR expression correlates with worse survival in MM, but this finding loses significance when histologic type (epithelioid vs. sarcomatoid) is taken into account (Dazzi, 1990). EGFR expression did not correlate with survival in a recent study (Destro, 2006).

We recently found that the activated nerve growth factor (NGF) receptor p-TrkA is more frequently expressed compared to p75, another NGF receptor that belongs to the tumor necrosis factor family, in MM, and that p-TrkA expression is significantly higher in peritoneal MM compared to their pleural counterparts (Figures 1-D, 1-E). In addition, p-TrkA expression was marginally higher in effusions, while p75 expression was significantly higher in solid MM (Davidson, 2004). This suggests that p-TrkA plays a significant role in the biology of this disease and may be a relevant therapeutic target, especially for MM cells in effusions.

We subsequently studied the anatomic site-related expression of angiogenic molecules in MM, including VEGF, IL-8, bFGF and heparanase. Heparanase is an endoglycosidase that degrades heparan sulfate in the extracellular matrix (ECM) and cell surfaces and has a role in cancer metastasis and angiogenesis (Edovitsky, 2004). We found significantly lower heparanase and bFGF expression in effusions compared with solid tumors (Davidson, 2004) (Figures 1-F, 1-G). This finding is comparable to our previous data in ovarian and breast carcinoma (Davidson, 2002b; Konstantinovsky, 2005) and suggests a reduced need for pro-angiogenic stimuli in effusions.

The above data suggest that multiple growth factor-initiated survival pathways may be activated in MM, although expression of some of these molecules may differ as function of the anatomic site and microenvironment. Studies investigating the therapeutic value of different receptor tyrosine kinase inhibitors are at advanced phase for many tumors, and inclusion of these agents in treatment protocols may be relevant for at least a subset of MM patients (Kindler, 2004).

## Apoptosis and Cell Cycle Molecules

As is the case for the majority of cancers, MM cells have deregulated response to pro-apoptotic signals and activate or deactivate cell cycle molecules that mediate sustained proliferation (reviewed in Fennell, 2004). Earlier work has shown that higher protein expression of the cell cycle inhibitor p27^kip1^ predicts longer survival in MM (Beer, 2001; Bongiovanni, 2001; Baldi, 2004), although this correlation was lost in multivariate analysis (Baldi, 2004). Protein expression of p21, another cell cycle inhibitor, correlated with better survival in univariate analysis in the latter study (Baldi, 2004), but showed no correlation with survival in an additional report (Isik, 2001). Interestingly, two independent studies found that both high proliferation and high apoptosis correlate with worse survival in MM (Beer, 2000; Kahlos, 2000), the latter report showing that higher levels of the anti-oxidant enzyme manganese superoxide dismutase are associated with low proliferation (Kahlos, 2000). Data from the same group show that the anti-apoptotic proteins Bcl-X and Mcl-1 and the pro-apoptotic protein Bax are co-expressed in MM, with less frequent expression of another anti-apoptotic protein, Bcl-2 (Soini, 1999). A recent study similarly showed co-expression of the anti-apoptotic proteins Bcl-2, Bcl-XL and Mcl-1, with variable loss of expression of the anti-apoptotic Bad, Bak, Bax, Bid and Bim (O’Kane, 2006). Expression of Fas ligand and loss of Bax were recently shown to correlate with shorter survival (Kokturk, 2005). Silencing methylation of TNF-related apoptosis-inducing ligand (TRAIL) receptors has been found in various cancers, including clinical specimens and MM cell lines, documenting an important mechanism for the evasion of apoptosis (Shivapurkar, 2004).

Recently, there has been growing focus on the role of the inhibitor of apoptosis (IAP) family in cancer. IAPs are caspase inhibitors that prevent apoptosis by specifically inhibiting caspases 3, 7 and 9. To date, eight human IAPs have been identified: cellular IAP1 (c-IAP1), cellular IAP2 (c-IAP2), neuronal apoptosis inhibitory protein (NAIP), Survivin, X- linked IAP (XIAP), Apollon, testis-specific IAP (Ts-IAP), and Livin (Nachmias, 2004). Frequent Survivin expression was found in MM cell lines and solid tumors, and blocking of Survivin by antisense oligonucleotides induced apoptosis in the MS-1 and H28 MM cell lines (Xia, 2002). Survivin levels decreased following treatment with Cisplatin, and treatment with anti-Survivin oligonucleotides resulted in p53 activation and sensitization to apoptosis in the ZL34 cell line (Hopkins-Donaldson, 2006). Survivin mRNA expression was found to be elevated in pleural MM and inflammatory pleuritis compared to normal pleura using real-time PCR (Falleni, 2005). c-IAP1 expression was reported to be elevated in pleural MM compared to normal pleura and lung tissue, and antisense targeting of cIAP-1 resulted in caspase 9 cleavage and sensitization to apoptosis in the 94–589 cell line (Gordon, 2002a).

We recently analyzed the expression of XIAP, Survivin and Livin in MM (Kleinberg, 2007). Using immunoblotting, we detected expression of XIAP and Survivin, but no expression of Livin, in effusion specimens. Immunohistochemical analysis of 112 MM showed significantly higher XIAP expression in peritoneal compared to pleural MM and in effusions compared to solid lesions, with reduced expression of nuclear (postulated proliferation-related) Survivin and the proliferation marker Ki-67 in effusions compared to solid tumors. These data suggest that XIAP and Survivin, but not Livin, are frequently expressed in MM, and that the reduced nuclear Survivin expression in effusions may be related to lesser degree of proliferation. The upregulation of XIAP in MM effusions and in peritoneal mesothelioma suggests a major pro-survival role for this IAP member at these anatomic sites.

These studies suggest that deregulation of cell death and survival by different mechanisms is frequent in MM, and that modulation of these pathways may be considered as a therapeutic modality in this cancer.

## Differentiation Markers

Two molecules that have received attention with respect to MM diagnosis and prognosis in recent years are the Wilms’ tumor 1 (WT1) gene and mesothelin. WT1 is a tumor suppressor gene localized to chromosome 11 that is inactivated through mutation in 10% of Wilms’ tumors, a pediatric malignancy of renal origin. However, it is expressed in other tumors, such as leukemia, desmoplastic small round cell tumor and MM, and may function as both tumor suppressor and survival factor (Scharnhorst, 2001). WT1 expression in MM correlates with expression of Syndecan-1 (Kumar-Singh 1998), but not of EGFR and IGFR, two transcriptional targets of WT1, or survival (Kumar-Singh, 1997). WT1 does seem to have a role in differentiating MM from lung and other AC, though not from the closely-related serous AC of the ovary, the main differential diagnosis of MM within the peritoneal cavity (Kumar-Singh, 1997).

Mesothelin is a 40-kDa cell surface glycoprotein that is synthesized from a 69-kDa precursor (Chang, 1996). Mesothelin binds to phosphatydilinositol, and dissociates from this signaling-related protein following treatment with phospholipase C (Chang, 1996). The biological role of mesothelin is uncertain at present, although it may have a role in cell adhesion (Chang, 1996). Mesothelin is frequently expressed on RM and MM, but despite earlier claims that its expression is limited to cells of mesothelial lineage (Urwin, 2000; Ordonez, 2003b), has been shown to be expressed in several tumor types, including non-mucinous ovarian, lung and pancreatic AC (Miettinen, 2003; Ordonez, 2003b). In view of the fact that lung and ovarian AC are two of the main differential diagnoses for MM, mesothelin appears to have limited value as a diagnostic marker. Mesothelin may yet have a role in other contexts. Serum mesothelin levels are elevated in MM, suggesting a role in early diagnosis of this tumor in high-risk populations (Robinson, 2003), a role as marker for monitoring treatment response (Hassan, 2006), and a potential target for tumor-related therapy (Hassan, 2004). A role in disease monitoring in MM was recently suggested also for megakaryocyte potentiation factor, a protein that is cleaved from a mesothelin precursor (Onda, 2006).

## Intracellular Signaling

Signals originating from cell surface receptors are relayed to the nucleus via intracellular networks that are predominantly regulated through activation of kinases and phosphatases. Molecular events that occur following exposure to asbestos *in vitro* have been shown to affect mitogen-activated protein kinase (MAPK) signaling (Zanella, 1996; Ramos-Nino, 2002; Shukla, 2003; Swain, 2004). Our group recently investigated protein expression (level) and phosphorylation status (activation) of the three MAPK members- extracellular-regulated kinase (ERK), c-Jun amino-terminal kinase (JNK) and high osmolarity glycerol response kinase (p38) in RM and MM specimens. Expression and activation of p38 (phospho-p38, p-p38) was found in the majority of specimens, with less frequent phosphorylation of ERK and JNK (Figure 1-H). MM and RM cells showed similar MAPK expression, activation and activation ratio. These data are the first evidence of *in vivo* activation of MAPK in clinical MM and RM. The similar values in these two groups suggest that MAPK may not be involved in the transformation of benign to malignant mesothelium, and therefore question the validity of MAPK as molecular therapeutic targets in MM (Vintman, 2005).

Recent work has shown that Wnt-1, member of the Wnt family, is involved in inhibition of apoptosis in MM *in vitro*, and that inhibition of Wnt-1 or its receptor Dishevelled induces apoptosis via JNK activation (You, 2004). These data are supported by the frequent inactivation through methylation of several secreted frizzled-related proteins, inhibitors of the Wnt pathway, in MM cell lines and clinical specimens (Lee, 2004).

Although these studies provide important data regarding intracellular signaling in MM, our understanding of these pathways in clinical MM is minimal at present, and their complexity warrants further research.

## The Immune Response

The host response in cancer involves the production of an array of molecules designed to inhibit cancer cell growth and metastasis. In reality, this process often achieves the opposite effect due to the ability of tumor cells to utilize host-produced molecules as growth promoters and to evade immune response-related mechanisms (Elenbaas, 2001).

Asbestos inhibits the activity of lymphocyte-activated killer cells (LAK) in vitro (Manning, 1991). Tumor-infiltrating lymphocytes show reduced expression of activation markers and the production of cytokines is dysregulated in a murine MM model (Bielefeldt-Ohmann, 1994). IL-12 and IL-2 have been shown to induce an anti-tumor effect in experimental models (Caminschi, 1998; Porta, 2000), while patient treatment with recombinant GM-CSF does not induce disease regression (Powell, 2006). However, anti-tumor effect was seen with use of soluble type II TGF-β receptor and interferon-β in experimental models (Odaka, 2001; Suzuki, 2004). Improved survival was observed following treatment of MM patients with Adeno-virus-mediated herpes simplex virus thymidine kinase/ganciclovir gene therapy (Sterman, 2005).

We recently studied two aspects of the immune response in MM. In the first study, we analyzed the expression of HLA-G, a non-classical major histocompatibility complex (MHC) class I that has been hypothesized to mediate cancer cell evasion of the host immune response (Algarra, 2004). We found that MM cells only rarely express HLA-G, but that expression is significantly higher in effusions (Figure 1-I), suggesting that cells at this site may utilize this surface molecule to evade the immune response in effusions (Kleinberg, 2006).

Chemokines are a family of small molecules that regulate the immune response and mediate several cancer-related events via specific receptors (Homey, 2002; Wilson, 2002). Chemokines produced by cancer and stromal cells attract lymphocytes and monocytes expressing their receptors to the tumor site. However, chemokine receptors have also been reported to be expressed on tumor cells, thereby creating an autocrine loop that mediates pro-growth signals (Homey, 2002). A recent study using a cytokine array system showed that the supernatant of cultured MM cells and the effusion fluid contain 25 different chemokines (Hegmans, 2006). We analyzed the characteristics of the leukocyte infiltrate expression and the expression of 5 chemokine receptors in MM and RM effusions. Chemokine receptors were frequently expressed on leukocytes in MM and RM effusions, but were infrequently present in MM cells and universally absent in RM cells. This finding suggests a major role for an autocrine chemokine pathway in leukocytes, but not in MM cells. We additionally found increased monocyte infiltration and monocyte chemokine receptor expression in MM compared to RM effusions, suggesting these cells may have a tumor-promoting rather than inhibiting effect (Davidson, 2007).

## High-Throughput Methods

Advances in molecular techniques allow for multi-parameter analysis of genes (comparative genomic hybridization, CGH), mRNA transcripts (cDNA arrays) and proteins (proteomics). Among these methods, cDNA analysis is the one that has been most often used in studies of MM. Comparative analysis of clinical specimens and MM cell lines identified candidate gene products that are upregulated in the former group, such as matriptase (Hoang, 2004b).

Comparative analysis of lung AC and pleural MM identified several markers that are differentially expressed by these two tumors, including calretinin and thyroid transcription factor-1 (TTF-1), that are commonly used in the diagnostic setting (Gordon, 2002b). Two studies in which MM specimens were compared to benign pleura and/or RM-derived cell lines have revealed a large number of genes that are differentially expressed in these two conditions (Singhal, 2003; Kettunen, 2005). Singhal et al. found 166 genes that were upregulated and 26 that were downregulated in MM, including cytoskeletal elements (e.g. annexins, integrins, keratins), molecules involved in protein synthesis and in metabolic pathways and gene products defined as having therapeutic and prognostic implications (Singhal, 2003). Kettunen et al. analyzed the expression of 588 gene products and found several that were over-expressed in MM compared to benign mesothelium (the collagen 1A2 chain and the β4 integrin subunit) and RM-derived cell lines (e.g. ezrin, bFGF, the tPA and uPA plasminogen activators, N-cadherin, cytokeratin 7), and others that were reduced in malignant cells (TRAIL, cytokeratin 19, α3 integrin subunit) (Kettunen, 2005). The latter study also reported histologic type-specific upregulation of gene products (e.g. P-cadherin in epithelioid MM; MMP-9 and tPA in sarcomatoid MM), thus providing molecular correlates for these morphologic differences (Kettunen, 2005). Two recent studies focused on the prognostic value of different molecules in MM (Gordon, 2003; Pass, 2004). Gordon et al. found several mRNAs that are expressed in higher levels in MM cases with better (e.g. hyaluronan synthase) and worse (e.g. insulin-like growth factor-binding protein-3; IGFBP-3) outcome (Gordon, 2003). Pass et al. found 27 genes with predictive value using two different statistical tests (dChip and SAM), including the α6 integrin subunit, the metastasis suppressor nm23, fibroblast growth factor 7 and IGFBP5 (Pass, 2004). A recent study by Lopez-Rios et al. identified several new genes that aid in the differentiation between sarcomatoid and epithelioid MM, including Uroplakin 1B and 3B, kallikrein 11, claudin 15 and Annexin A9, all more highly expressed in epithelioid MM (Lopez-Rios, 2006). Of note, the authors report relatively low predictive value for survival for this method, with no significant additive power to data obtained by standard clinicopathologic variables and p16/CDNK2A status, suggesting that global gene expression profiling may have a greater role as a research tool than in clinical practice (Lopez-Rios, 2006).

Along this line, our group recently performed a cDNA analysis comparing diffuse malignant peritoneal mesothelioma (DMPM) and ovarian carcinoma cells in effusions, two tumors with common histogenesis that share expression of many diagnostic and differentiation markers. In this analysis, we identified 189 genes that are differentially expressed in these cancers, including higher gene expression of calretinin, vitronectin, claudin 15, α4 laminin and hyaluronan synthase 1 in DMPM, and higher IGF-II, IGFBP-3, cyclin E1, folate receptors 1 and 3, RAB25, MUC4, endothelin-1, CD24, kallikreins 6/7/8, claudins 3/4/6, Notch3 and MMP-7 expression in ovarian carcinoma (Davidson, 2006).

How this novel knowledge will impact on the diagnosis and clinical management of MM is yet to be seen. One area that has not yet received sufficient attention is large-scale protein analysis. Although data on mRNA level reflect the transcriptional activity of MM cells, protein analysis is more directly related to the biological activity of these molecules. Hegmans et al. recently studied the protein profile of exosomes, small membrane vesicles that are secreted into the ECM, in MM cell lines using the matrix-assisted laser desorption ionization time-of-flight (MALDI-TOF). The authors identified several proteins that are secreted by MM cells *in vitro*, including MHC class I antigens, heat shock proteins (HSC70, HSP90), and cytoskeletal proteins (ezrin, actinin-4) (Hegmans, 2004). If confirmed in clinical material, these proteins may teach us more about the microenvironment of MM and provide new molecular targets for directed therapy.

## Concluding Remarks

The rise in the incidence of MM presents a growing health problem and emphasizes the need for improved diagnosis, prognostication and treatment in this cancer. The body of work reviewed in this paper documents the growing effort to understand the biology of this disease and its genetic make-up, and to define the biological characteristics that favor the survival of MM cells. Our own data reveal differences between peritoneal and pleural MM, and between MM cells in solid lesions and effusions, suggesting that the tumor microenvironment in part regulates the synthesis of cancer-associated molecules in MM.

To date, our understanding of MM biology and the efforts to target key-molecules in this disease have not significantly altered the poor survival associated with this cancer type. However, knowledge regarding the biology of MM is being more frequently translated into new treatment approaches. Vogelzang et al. recently reviewed new agents that under evaluation for the treatment of MM, including EGFR, PDGFR, VEGF and HGF inhibitors, inhibitors of mTOR, a downstream molecule of the PI3K/AKT pathway, and inhibitors of the proteasome/ubiquitin pathway (Vogelzang, 2005). Although recent results in clinical trials using the tyrosine kinase receptor inhibitor imatinib mesylate, which targets PDGFR and c-Kit, have not been encouraging (Mathy, 2005; Porta, 2007), it is to be hoped that the common effort of medical and research disciplines will in the future allow us to achieve more success in treating this highly lethal tumor.

## References

[b1-bmi-2007-173] Abutaily AS Collins JE Roche WR 2003 Cadherins, catenins and APC in pleural malignant mesothelioma J Pathol 201 355 62 14595746 10.1002/path.1458

[b2-bmi-2007-173] Algarra I Garcia-Lora A Cabrera T 2004 The selection of tumor variants with altered expression of classical and nonclassical MHC class I molecules: implications for tumor immune escape Cancer Immunol Immunother 53 904 10 15069585 10.1007/s00262-004-0517-9 PMC11032983

[b3-bmi-2007-173] Ascoli V Scalzo CC Facciolo F 1995 Platelet-derived growth factor receptor immunoreactivity in mesothelioma and nonneoplastic mesothelial cells in serous effusions Acta Cytol 39 613 22 7543232

[b4-bmi-2007-173] Baldi A Groeger AM Esposito V 2002 Expression of p21 in SV40 large T antigen positive human pleural mesothelioma: relationship with survival Thorax 57 353 6 11923556 10.1136/thorax.57.4.353 PMC1746306

[b5-bmi-2007-173] Baldi A Santini D Vasaturo F 2004 Prognostic significance of cyclooxygenase-2 (COX-2) and expression of cell cycle inhibitors p21 and p27 in human pleural malignant mesothelioma Thorax 59 428 33 15115874 10.1136/thx.2003.008912 PMC1746992

[b6-bmi-2007-173] Bedrossian CWM 1994 Malignant effusions: A multimodal approach to cytologic diagnosis New-York Igaku-Shoin

[b7-bmi-2007-173] Bedrossian CW 1998 Special stains, the old and the new: the impact of immunocytochemistry in effusion cytology Diagn Cytopathol 18 141 9 9484644 10.1002/(sici)1097-0339(199802)18:2<141::aid-dc11>3.0.co;2-l

[b8-bmi-2007-173] Beer TW Carr NJ Whittaker MA 2000 Mitotic and in situ end-labeling apoptotic indices as prognostic markers in malignant mesothelioma Ann Diagn Pathol 4 143 8 10919383 10.1016/s1092-9134(00)90036-4

[b9-bmi-2007-173] Beer TW Shepherd P Pullinger NC 2001 p27 immunostaining is related to prognosis in malignant mesothelioma Histopathology 38 535 41 11422497 10.1046/j.1365-2559.2001.01136.x

[b10-bmi-2007-173] Bianchi AB Mitsunaga SI Cheng JQ 1995 High frequency of inactivating mutations in the neurofibromatosis type 2 gene (NF2) in primary malignant mesotheliomas Proc Natl Acad Sci USA 92 10854 8 7479897 10.1073/pnas.92.24.10854 PMC40529

[b11-bmi-2007-173] Bielefeldt-Ohmann H Fitzpatrick DR Marzo AL 1994 Patho- and immunobiology of malignant mesothelioma: characterisation of tumour infiltrating leucocytes and cytokine production in a murine model Cancer Immunol Immunother 39 347 59 8001022 10.1007/BF01534421 PMC11041107

[b12-bmi-2007-173] Bongiovanni M Cassoni P De Giuli P 2001 p27(kip1) immuno-reactivity correlates with long-term survival in pleural malignant mesothelioma Cancer 92 1245 50 11571739 10.1002/1097-0142(20010901)92:5<1245::aid-cncr1444>3.0.co;2-g

[b13-bmi-2007-173] Brittonm M 2002 The epidemiology of mesothelioma Semin Oncol 29 18 25 10.1053/sonc.2002.30237 11836665

[b14-bmi-2007-173] Burgers JA Damhuis RA 2004 Prognostic factors in malignant mesothelioma Lung Cancer, 45 Suppl 1 S49 54 10.1016/j.lungcan.2004.04.012 15261434

[b15-bmi-2007-173] Cacciotti P Libener R Betta P 2001 SV40 replication in human mesothelial cells induces HGF/Met receptor activation: a model for viral-related carcinogenesis of human malignant mesothelioma Proc Natl Acad Sci USA 98 12032 7 11572935 10.1073/pnas.211026798 PMC59762

[b16-bmi-2007-173] Cacciotti P Strizzi L Vianale G 2002 The presence of simian-virus 40 sequences in mesothelioma and mesothelial cells is associated with high levels of vascular endothelial growth factor Am J Respir Cell Mol Biol 26 189 93 11804869 10.1165/ajrcmb.26.2.4673

[b17-bmi-2007-173] Cacciotti P Barbone D Porta C 2005 SV40-dependent AKT activity drives mesothelial cell transformation after asbestos exposure Cancer Res 65 5256 62 15958571 10.1158/0008-5472.CAN-05-0127

[b18-bmi-2007-173] Cai YC Roggli V Mark E Cagle PT 2004 Transforming growth factor alpha and epidermal growth factor receptor in reactive and malignant mesothelial proliferations Arch Pathol Lab Med 128 68 70 14692808 10.5858/2004-128-68-TGFAEG

[b19-bmi-2007-173] Caminschi I Venetsanakos E Leong CC 1998 Interleukin-12 induces an effective antitumor response in malignant mesothelioma Am J Respir Cell Mol Biol 19 738 46 9806738 10.1165/ajrcmb.19.5.3257m

[b20-bmi-2007-173] Carbone M Rudzinski J Bocchetta M 2003 High throughput testing of the SV40 Large T antigen binding to cellular p53 identifies putative drugs for the treatment of SV40-related cancers Virology 315 409 14 14585344 10.1016/s0042-6822(03)00547-6

[b21-bmi-2007-173] Catalano A Romano M Martinotti S 2002 Enhanced expression of vascular endothelial growth factor (VEGF) plays a critical role in the tumor progression potential induced by simian virus 40 large T cell antigen Oncogene 21 2896 900 11973650 10.1038/sj.onc.1205382

[b22-bmi-2007-173] Chang K Pastan I 1996 Molecular cloning of mesothelin, a differentiation antigen present on mesothelium, mesotheliomas, and ovarian cancers Proc Natl Acad Sci USA 93 136 40 8552591 10.1073/pnas.93.1.136 PMC40193

[b23-bmi-2007-173] Cote RJ Jhanwar SC Novick S 1991 Genetic alterations of the p53 gene are a feature of malignant mesotheliomas Cancer Res 51 5410 6 1913660

[b24-bmi-2007-173] Davidson B Nielsen S Christensen J 2001 The role of Desmin and N-cadherin in effusion cytology. A comparative study using established markers of mesothelial and epithelial cells Am J Surg Pathol 25 1405 12 11684957 10.1097/00000478-200111000-00008

[b25-bmi-2007-173] Davidson B Dong HP Berner A 2002 Detection of malignant epithelial cells in effusions using Flow cytometric immunophenotyping- an analysis of 92 cases Am J Clin Pathol 118 85 92 12109861 10.1309/M877-QABM-D9GB-FJAX

[b26-bmi-2007-173] Davidson B Reich R Kopolovic J 2002 Interleukin-8 and vascular endothelial growth factor mRNA levels are down-regulated in ovarian carcinoma cells in serous effusions Clin Exp Metastasis 19 135 44 11964077 10.1023/a:1014582911680

[b27-bmi-2007-173] Davidson B Reich R Lazarovici P 2004 Expression of the nerve growth factor receptors TrkA and p75 in malignant mesothelioma Lung Cancer 44 159 65 15084380 10.1016/j.lungcan.2003.11.014

[b28-bmi-2007-173] Davidson B Vintman L Zcharia E 2004 Heparanase and basic fibroblast growth factor are co-expressed in malignant mesothelioma Clin Exp Metastasis 21 469 76 15672872 10.1007/s10585-004-3150-2

[b29-bmi-2007-173] Davidson B Zhang Z Kleinberg L 2006 Gene expression signatures differentiate ovarian/peritoneal serous carcinoma from diffuse malignant peritoneal mesothelioma Clin Cancer Res 12 5944 50 17062665 10.1158/1078-0432.CCR-06-1059

[b30-bmi-2007-173] Davidson B Dong HP Holth A 2007 Chemokine receptors are infrequently expressed in malignant or benign mesothelial cells In press Am J Clin Pathol 127 752 9 17439834 10.1309/LN2075V7C8K31CH8

[b31-bmi-2007-173] Dazzi H Hasleton PS Thatcher N 1990 Malignant pleural mesothelioma and epidermal growth factor receptor (EGF-R). Relationship of EGF-R with histology and survival using fixed paraffin embedded tissue and the F4, monoclonal antibody Br J Cancer 61 924 6 2372497 10.1038/bjc.1990.207 PMC1971675

[b32-bmi-2007-173] Dedhar S 2000 Cell-substrate interactions and signaling through ILK Curr Opin Cell Biol 12 250 6 10712922 10.1016/s0955-0674(99)00083-6

[b33-bmi-2007-173] Destro A Ceresoli GL Falleni M 2006 EGFR overexpression in malignant pleural mesothelioma. An immunohistochemical and molecular study with clinicopathological correlations Lung Cancer 51 207 15 16384623 10.1016/j.lungcan.2005.10.016

[b34-bmi-2007-173] De Wever O Mareel M 2003 Role of tissue stroma in cancer cell invasion J Pathol 200 429 47 12845611 10.1002/path.1398

[b35-bmi-2007-173] Dorai T Kobayashi H Holland JF 1994 Modulation of platelet-derived growth factor-beta mRNA expression and cell growth in a human mesothelioma cell line by a hammerhead ribozyme Mol Pharmacol 46 437 44 7935323

[b36-bmi-2007-173] Edovitsky E Elkin M Zcharia E 2004 Heparanase gene silencing, tumor invasiveness, angiogenesis, and metastasis J Natl Cancer Inst 96 1219 30 15316057 10.1093/jnci/djh230

[b37-bmi-2007-173] Edwards JG Cox G Andi A 2001 Angiogenesis is an independent prognostic factor in malignant mesothelioma Br J Cancer 85 863 8 11556838 10.1054/bjoc.2001.1997 PMC2375086

[b38-bmi-2007-173] Edwards JG McLaren J Jones JL 2003 Matrix metalloproteinases 2 and 9 (gelatinases A and B) expression in malignant mesothelioma and benign pleura Br J Cancer 88 1553 9 12771921 10.1038/sj.bjc.6600920 PMC2377107

[b39-bmi-2007-173] Egeblad M Werb Z 2002 New functions for the matrix metalloproteinases in cancer progression Nature Rev Cancer 2 161 74 11990853 10.1038/nrc745

[b40-bmi-2007-173] Elenbaas B Weinberg RA 2001 Heterotypic signaling between epithelial tumor cells and fibroblasts in carcinoma formation Exp Cell Res 264 169 84 11237532 10.1006/excr.2000.5133

[b41-bmi-2007-173] Eltabbakh GH Piver MS Hempling RE 1999 Clinical picture, response to therapy, and survival of women with diffuse malignant peritoneal mesothelioma J Surg Oncol 70 6 12 9989414 10.1002/(sici)1096-9098(199901)70:1<6::aid-jso2>3.0.co;2-x

[b42-bmi-2007-173] Falleni M Pellegrini C Marchetti A 2005 Quantitative evaluation of the apoptosis regulating genes Survivin, Bcl-2 and Bax in inflammatory and malignant pleural lesions Lung Cancer 48 211 6 15829320 10.1016/j.lungcan.2004.10.003

[b43-bmi-2007-173] Fennell DA Rudd RM 2004 Defective core-apoptosis signalling in diffuse malignant pleural mesothelioma: opportunities for effective drug development Lancet Oncol 5 354 62 15172356 10.1016/S1470-2045(04)01492-5

[b44-bmi-2007-173] Galffy G Mohammed KA Dowling PA 1999 Interleukin 8: An autocrine growth factor for malignant mesothelioma Cancer Res 59 367 71 9927048

[b45-bmi-2007-173] Foddis R De Rienzo A Broccoli D 2002 SV40 infection induces telomerase activity in human mesothelial cells Oncogene 21 1434 42 11857086 10.1038/sj.onc.1205203

[b46-bmi-2007-173] Givant-Horwitz V Davidson B Reich R 2004 Laminin-induced signaling in tumor cells: the role of the M(r) 67,000 laminin receptor Cancer Res 64 3572 9 15150114 10.1158/0008-5472.CAN-03-3424

[b47-bmi-2007-173] Gordon GJ Appasani K Parcells JP 2002 Inhibitor of apoptosis protein-1 promotes tumor cell survival in mesothelioma Carcinogenesis 23 1017 24 12082024 10.1093/carcin/23.6.1017

[b48-bmi-2007-173] Gordon GJ Jensen RV Hsiao LL 2002 Translation of microarray data into clinically relevant cancer diagnostic tests using gene expression ratios in lung cancer and mesothelioma Cancer Res 62 4963 7 12208747

[b49-bmi-2007-173] Gordon GJ Jensen RV Hsiao LL 2003 Using gene expression ratios to predict outcome among patients with mesothelioma J Natl Cancer Inst 95 598 605 12697852 10.1093/jnci/95.8.598

[b50-bmi-2007-173] Gulyas M Hjerpe A 2003 Proteoglycans and WT1 as markers for distinguishing adenocarcinoma, epithelioid mesothelioma, and benign mesothelium J Pathol 199 479 87 12635139 10.1002/path.1312

[b51-bmi-2007-173] Han AC Peralta-Soler A Knudsen KA 1997 Differential expression of N-cadherin in pleural mesotheliomas and E-cadherin in lung adenocarcinomas in formalin-fixed, paraffin-embedded tissues Hum Pathol 28 641 5 9190996 10.1016/s0046-8177(97)90171-4

[b52-bmi-2007-173] Hanahan D Weinberg RA 2000 The hallmarks of cancer Cell 2000 100 57 70 10647931 10.1016/s0092-8674(00)81683-9

[b53-bmi-2007-173] Harvey P Clark IM Jaurand MC 2000 Hepatocyte growth factor/scatter factor enhances the invasion of mesothelioma cell lines and the expression of matrix metalloproteinases Br J Cancer 83 1147 53 11027427 10.1054/bjoc.2000.1445 PMC2363594

[b54-bmi-2007-173] Hassan R Bera T Pastan I 2004 Mesothelin: a new target for immunotherapy Clin Cancer Res 10 3937 42 15217923 10.1158/1078-0432.CCR-03-0801

[b55-bmi-2007-173] Hassan R Remaley AT Sampson ML 2006 Detection and quantification of serum mesothelin, a tumor marker for patients with mesothelioma and ovarian cancer Clin Cancer Res 12 447 53 16428485 10.1158/1078-0432.CCR-05-1477

[b56-bmi-2007-173] Hegmans JP Bard MP Hemmes A 2004 Proteomic analysis of exosomes secreted by human mesothelioma cells Am J Pathol 164 1807 15 15111327 10.1016/S0002-9440(10)63739-X PMC1615654

[b57-bmi-2007-173] Hegmans JP Hemmes A Hammad H 2006 Mesothelioma environment comprises cytokines and T-regulatory cells that suppress immune responses Eur Respir J 27 1086 95 16540497 10.1183/09031936.06.00135305

[b58-bmi-2007-173] Hoang CD Zhang X Scott PD 2004 Selective activation of insulin receptor substrate-1 and -2 in pleural mesothelioma cells: association with distinct malignant phenotypes Cancer Res 64 7479 85 15492273 10.1158/0008-5472.CAN-04-1898

[b59-bmi-2007-173] Hoang CD D’Cunha J Kratzke MG 2004 Gene expression profiling identifies matriptase overexpression in malignant mesothelioma Chest 125 1843 52 15136399 10.1378/chest.125.5.1843

[b60-bmi-2007-173] Homey B Müller A Zlotnik A 2002 Chemokines: agents for immunotherapy of cancer Nature Rev Immunol 2 175 84 11913068 10.1038/nri748

[b61-bmi-2007-173] Hopkins-Donaldson S Belyanskaya LL Simoes-Wust AP 2006 p53-induced apoptosis occurs in the absence of p14(ARF) in malignant pleural mesothelioma Neoplasia 8 551 9 16867217 10.1593/neo.06148 PMC1601933

[b62-bmi-2007-173] Howe A Aplin AE Alahari SK 1998 Integrin signaling and cell growth control Curr Opin Cell Biol 10 220 31 9561846 10.1016/s0955-0674(98)80144-0

[b63-bmi-2007-173] Hynes RO 1992 Integrins: versatility, modulation, and signaling in cell adhesion Cell 69 11 25 1555235 10.1016/0092-8674(92)90115-s

[b64-bmi-2007-173] Illei PB Rusch VW Zakowski MF 2003 Homozygous deletion of CDKN2A and codeletion of the methylthioadenosine phosphorylase gene in the majority of pleural mesotheliomas Clin Cancer Res 9 2108 13 12796375

[b65-bmi-2007-173] Illei PB Ladanyi M Rusch VW 2003 The use of CDKN2A deletion as a diagnostic marker for malignant mesothelioma in body cavity effusions Cancer Cytopathol 99 51 6 10.1002/cncr.10923 12589646

[b66-bmi-2007-173] Isik R Metintas M Gibbs AR 2001 p53, p21 and metallothionein immunoreactivities in patients with malignant pleural mesothelioma: correlations with the epidemiological features and prognosis of mesotheliomas with environmental asbestos exposure Respir Med 95 588 93 11453316 10.1053/rmed.2001.1108

[b67-bmi-2007-173] Kahlos K Soini Y Paakko P 2000 Proliferation, apoptosis, and manganese superoxide dismutase in malignant mesothelioma Int J Cancer 88 37 43 10962437 10.1002/1097-0215(20001001)88:1<37::aid-ijc6>3.0.co;2-3

[b68-bmi-2007-173] Kettunen E Nicholson AG Nagy B 2005 L1CAM, INP10, P-cadherin, tPA and ITGB4 over-expression in malignant pleural mesotheliomas revealed by combined use of cDNA and tissue microarray Carcinogenesis 26 17 25 15447976 10.1093/carcin/bgh276

[b69-bmi-2007-173] Kindler HL 2004 Moving beyond chemotherapy: novel cytostatic agents for malignant mesothelioma Lung Cancer 45 Suppl 1 S125 7 15261445 10.1016/j.lungcan.2004.04.022

[b70-bmi-2007-173] Kleinberg L Flørenes VA Skrede M 2006 Expression of HLA-G in malignant mesothelioma and clinically aggressive breast carcinoma Virchows Arch 449 31 9 16541284 10.1007/s00428-005-0144-7

[b71-bmi-2007-173] Kleinberg L Lie AK Flørenes VA 2007 Expression of inhibitors of apoptosis (IAP) family members in malignant mesothelioma In press Hum Pathol [Epub ahead of print] 10.1016/j.humpath.2006.12.013 17350081

[b72-bmi-2007-173] Klominek J Sumitran Karuppan S Hauzenberger D 1997 Differential motile response of human malignant mesothelioma cells to fibronectin, laminin and collagen type IV: the role of beta1 integrins Int J Cancer 72 1034 44 9378538 10.1002/(sici)1097-0215(19970917)72:6<1034::aid-ijc19>3.0.co;2-4

[b73-bmi-2007-173] Klominek J Baskin B Hauzenberger D 1998 Platelet-derived growth factor (PDGF) BB acts as a chemoattractant for human malignant mesothelioma cells via PDGF receptor beta-integrin alpha3beta1 interaction Clin Exp Metastasis 16 529 39 9872600 10.1023/a:1006542301794

[b74-bmi-2007-173] Klominek J Baskin B Liu Z 1998 Hepatocyte growth factor/scatter factor stimulates chemotaxis and growth of malignant mesothelioma cells through c-met receptor Int J Cancer 76 240 9 9537587 10.1002/(sici)1097-0215(19980413)76:2<240::aid-ijc12>3.0.co;2-g

[b75-bmi-2007-173] Kokturk N Firat P Akay H 2005 Prognostic significance of Bax and Fas ligand in erionite and asbestos induced Turkish malignant pleural mesothelioma Lung Cancer 50 189 98 16043260 10.1016/j.lungcan.2005.05.025

[b76-bmi-2007-173] Konig JE Tolnay E Wiethege T 1999 Expression of vascular endothelial growth factor in diffuse malignant pleural mesothelioma Virchows Arch 435 8 12 10431840 10.1007/s004280050388

[b77-bmi-2007-173] Konig J Tolnay E Wiethege T 2000 Co-expression of vascular endothelial growth factor and its receptor flt-1 in malignant pleural mesothelioma Respiration 67 36 40 10705260 10.1159/000029460

[b78-bmi-2007-173] Konstantinovsky S Nielsen S Vyberg M 2005 Angiogenic molecule expression is downregulated in effusions from breast cancer patients Breast Cancer Res Treat 94 71 80 16142438 10.1007/s10549-005-7328-3

[b79-bmi-2007-173] Koukoulis GK Shen J Monson R 1997 Pleural mesotheliomas have an integrin profile distinct from visceral carcinomas Hum Pathol 28 84 90 9013837 10.1016/s0046-8177(97)90284-7

[b80-bmi-2007-173] Kumar-Singh S Segers K Rodeck U 1997 WT1 mutation in malignant mesothelioma and WT1 immunoreactivity in relation to p53 and growth factor receptor expression, cell-type transition, and prognosis J Pathol 181 67 74 9072005 10.1002/(SICI)1096-9896(199701)181:1<67::AID-PATH723>3.0.CO;2-Z

[b81-bmi-2007-173] Kumar-Singh S Jacobs W Dhaene K 1998 Syndecan-1 expression in malignant mesothelioma: correlation with cell differentiation, WT1 expression, and clinical outcome J Pathol 186 300 5 10211120 10.1002/(SICI)1096-9896(1998110)186:3<300::AID-PATH180>3.0.CO;2-Q

[b82-bmi-2007-173] Kumar-Singh S Weyler J Martin MJH 1999 Angiogenic cytokines in mesothelioma: A study of VEGF, FGF-1 and -2, and TGFβ expression J Pathol 189 72 8 10451491 10.1002/(SICI)1096-9896(199909)189:1<72::AID-PATH401>3.0.CO;2-0

[b83-bmi-2007-173] Langerak AW De Laat PA Van Der Linden-Van Beurden CA 1996 Expression of platelet-derived growth factor (PDGF) and PDGF receptors in human malignant mesothelioma in vitro and in vivo J Pathol 178 151 60 8683381 10.1002/(SICI)1096-9896(199602)178:2<151::AID-PATH425>3.0.CO;2-E

[b84-bmi-2007-173] Lantuejoul S Laverriere MH Sturm N 2000 NCAM (neural cell adhesion molecules) expression in malignant mesotheliomas Hum Pathol 31 415 21 10821486 10.1053/hp.2000.6552

[b85-bmi-2007-173] Lee AY He B You L 2004 Expression of the secreted frizzled-related protein gene family is downregulated in human mesothelioma Oncogene 23 6672 6 15221014 10.1038/sj.onc.1207881

[b86-bmi-2007-173] Liu Z Ivanoff A Klominek J 2001 Expression and activity of matrix metalloproteases in human malignant mesothelioma cell lines Int J Cancer 91 638 43 11267973 10.1002/1097-0215(200002)9999:9999<::aid-ijc1102>3.0.co;2-y

[b87-bmi-2007-173] Liu Z Klominek J 2003 Regulation of matrix metalloprotease activity in malignant mesothelioma cell lines by growth factors Thorax 58 198 203 12612292 10.1136/thorax.58.3.198 PMC1746590

[b88-bmi-2007-173] Lopez-Rios F Illei PB Rusch V 2004 Evidence against a role for SV40 infection in human mesotheliomas and high risk of false-positive PCR results owing to presence of SV40 sequences in common laboratory plasmids Lancet 364 1157 66 15451223 10.1016/S0140-6736(04)17102-X

[b89-bmi-2007-173] Lopez-Rios F Chuai S Flores R 2006 Global gene expression profiling of pleural mesotheliomas: overexpression of aurora kinases and p16/CDKN2A deletion as prognostic factors and critical evaluation of microarray-based prognostic prediction Cancer Res 66 2970 9 16540645 10.1158/0008-5472.CAN-05-3907

[b90-bmi-2007-173] Manfredi JJ Dong J Liu WJ 2005 Evidence against a role for SV40 in human mesothelioma Cancer Res 65 2602 9 15805256 10.1158/0008-5472.CAN-04-2461

[b91-bmi-2007-173] Manning LS Davis MR Robinson BW 1991 Asbestos fibres inhibit the in vitro activity of lymphokine-activated killer (LAK) cells from healthy individuals and patients with malignant mesothelioma Clin Exp Immunol 83 85 91 1846329 10.1111/j.1365-2249.1991.tb05593.x PMC1535470

[b92-bmi-2007-173] Manning CB Cummins AB Jung MW 2002 A mutant epidermal growth factor receptor targeted to lung epithelium inhibits asbestos-induced proliferation and proto-oncogene expression Cancer Res 62 4169 75 12154012

[b93-bmi-2007-173] Mathy A Baas P Dalesio O 2005 Limited efficacy of imatinib mesylate in malignant mesothelioma: a phase II trial Lung Cancer 50 83 6 15951053 10.1016/j.lungcan.2005.04.010

[b94-bmi-2007-173] Miettinen M Sarlomo-Rikala M 2003 Expression of calretinin, thrombomodulin, keratin 5, and mesothelin in lung carcinomas of different types: an immunohistochemical analysis of 596 tumors in comparison with epithelioid mesotheliomas of the pleura Am J Surg Pathol 27 150 8 12548160 10.1097/00000478-200302000-00002

[b95-bmi-2007-173] Moskal TL Urschel JD Anderson TM 1998 Malignant pleural mesothelioma: a problematic review Surg Oncol 7 5 12 10421502 10.1016/s0960-7404(98)00019-x

[b96-bmi-2007-173] Musti M Kettunen E Dragonieri S 2006 Cytogenetic and molecular genetic changes in malignant mesothelioma Cancer Genet Cytogenet 170 9 15 16965949 10.1016/j.cancergencyto.2006.04.011

[b97-bmi-2007-173] Nachmias B Ashhab Y Ben-Yehuda D 2004 The inhibitor of apoptosis protein family (IAPs): an emerging therapeutic target in cancer Semin Cancer Biol 14 231 43 15219616 10.1016/j.semcancer.2004.04.002

[b98-bmi-2007-173] Nagel H Schulten HJ Gunawan B 2002 The potential value of comparative genomic hybridization analysis in effusion-and fine needle aspiration cytology Mod Pathol 15 818 25 12181266 10.1097/01.MP.0000024521.67720.0F

[b99-bmi-2007-173] Odaka M Sterman DH Wiewrodt R 2001 Eradication of intraperitoneal and distant tumor by adenovirus-mediated interferon-beta gene therapy is attributable to induction of systemic immunity Cancer Res 61 6201 12 11507073

[b100-bmi-2007-173] Ohta Y Shridhar V Bright RK 1999 VEGF and VEGF type C play an important role in angiogenesis and lymphangiogenesis in human malignant mesothelioma tumours Br J Cancer 81 54 61 10487612 10.1038/sj.bjc.6690650 PMC2374345

[b101-bmi-2007-173] Ohta Y Shridhar V Kalemkerian GP 1999 Thrombospondin-1 expression and clinical implications in malignant pleural mesothelioma Cancer 85 2570 6 10375104

[b102-bmi-2007-173] O’Kane SL Pound RJ Campbell A 2006 Expression of bcl-2 family members in malignant pleural mesothelioma Acta Oncol 45 449 53 16760181 10.1080/02841860500468927

[b103-bmi-2007-173] Onda M Nagata S Ho M 2006 Megakaryocyte potentiation factor cleaved from mesothelin precursor is a useful tumor marker in the serum of patients with mesothelioma Clin Cancer Res 12 4225 31 16857795 10.1158/1078-0432.CCR-06-0472

[b104-bmi-2007-173] Ordonez NG 2003 Value of E-cadherin and N-cadherin immunostaining in the diagnosis of mesothelioma Hum Pathol 34 749 55 14506634 10.1016/s0046-8177(03)00285-5

[b105-bmi-2007-173] Ordonez NG 2003 Application of mesothelin immunostaining in tumor diagnosis Am J Surg Pathol 27 1418 28 14576474 10.1097/00000478-200311000-00003

[b106-bmi-2007-173] Pache JC Janssen YM Walsh ES 1998 Increased epidermal growth factor-receptor protein in a human mesothelial cell line in response to long asbestos fibers Am J Pathol 152 333 40 9466557 PMC1857975

[b107-bmi-2007-173] Parker C Neville E Lung cancer 2003 8: management of malignant mesothelioma Thorax 58 809 13 12947146 10.1136/thorax.58.9.809 PMC1746789

[b108-bmi-2007-173] Pass HI Pogrebniak HW 1993 Malignant pleural mesothelioma Curr Probl Surg 30 921 1012 8404013 10.1016/0011-3840(93)90029-g

[b109-bmi-2007-173] Pass HI Liu Z Wali A Bueno R 2004 Gene expression profiles predict survival and progression of pleural mesothelioma Clin Cancer Res 10 849 59 14871960 10.1158/1078-0432.ccr-0607-3

[b110-bmi-2007-173] Porta C Danova M Orengo AM 2000 Interleukin-2 induces cell cycle perturbations leading to cell growth inhibition and death in malignant mesothelioma cells in vitro J Cell Physiol 185 126 34 10942526 10.1002/1097-4652(200010)185:1<126::AID-JCP12>3.0.CO;2-2

[b111-bmi-2007-173] Porta C Mutti L Tassi G 2007 Negative results of an Italian Group for Mesothelioma (G.I.Me.) pilot study of single-agent imatinib mesylate in malignant pleural mesothelioma Cancer Chemother Pharmacol 59 149 50 16636799 10.1007/s00280-006-0243-4

[b112-bmi-2007-173] Powell A Creaney J Broomfield S 2006 Recombinant GM-CSF plus autologous tumor cells as a vaccine for patients with mesothelioma Lung Cancer 52 189 97 16563560 10.1016/j.lungcan.2006.01.007

[b113-bmi-2007-173] Prins JB Williamson KA Kamp MM 1998 The gene for the cyclin-dependent-kinase-4 inhibitor, CDKN2A, is preferentially deleted in malignant mesothelioma Int J Cancer 75 649 53 9466670 10.1002/(sici)1097-0215(19980209)75:4<649::aid-ijc25>3.0.co;2-2

[b114-bmi-2007-173] Pylkkanen L Wolff H Stjernvall T 2004 Reduced Fhit protein expression in human malignant mesothelioma Virchows Arch 444 43 8 14569398 10.1007/s00428-003-0902-3

[b115-bmi-2007-173] Ramael M Buysse C van den Bossche J 1992 Immunoreactivity for the beta chain of the platelet-derived growth factor receptor in malignant mesothelioma and non-neoplastic mesothelium J Pathol 167 1 4 1320670 10.1002/path.1711670102

[b116-bmi-2007-173] Ramos-Nino ME Timblin CR Mossman BT 2002 Mesothelial cell transformation requires increased AP-1 binding activity and ERK-dependent Fra-1 expression Cancer Res 62 6065 9 12414630

[b117-bmi-2007-173] Ramos-Nino ME Vianale G Sabo-Attwood T 2005 Human mesothelioma cells exhibit tumor cell-specific differences in phosphatidylinositol 3-kinase/AKT activity that predict the efficacy of Onconase Mol Cancer Ther 4 835 42 15897248 10.1158/1535-7163.MCT-04-0243

[b118-bmi-2007-173] Reich R Vintman L Nielsen S 2005 Differential expression of the 67 kDa laminin receptor in malignant mesothelioma and carcinomas that spread to serosal cavities Diagn Cytopathol 33 332 7 16240397 10.1002/dc.20296

[b119-bmi-2007-173] Robinson BW Creaney J Lake R 2003 Mesothelin-family proteins and diagnosis of mesothelioma Lancet 362 1612 6 14630441 10.1016/S0140-6736(03)14794-0

[b120-bmi-2007-173] Ruoslahti E 1991 Integrins J Clin Invest 87 1 5 1985087 10.1172/JCI114957 PMC294975

[b121-bmi-2007-173] Sandberg AA Bridge JA 2001 Updates on the cytogenetics and molecular genetics of bone and soft tissue tumors. Mesothelioma Cancer Genet Cytogenet 127 93 110 11425448 10.1007/3-540-30792-3_7

[b122-bmi-2007-173] Scharnhorst V van der Eb AJ Jochemsen AG 2001 WT1 proteins: functions in growth and differentiation Gene 273 141 61 11595161 10.1016/s0378-1119(01)00593-5

[b123-bmi-2007-173] Sekido Y Pass HI Bader S 1995 Neurofibromatosis type 2 (NF2) gene is somatically mutated in mesothelioma but not in lung cancer Cancer Res 55 1227 31 7882313

[b124-bmi-2007-173] Shah KV 2007 SV40 and human cancer: a review of recent data Int J Cancer 120 215 23 17131333 10.1002/ijc.22425

[b125-bmi-2007-173] Shivapurkar N Toyooka S Toyooka KO 2004 Aberrant methylation of trail decoy receptor genes is frequent in multiple tumor types Int J Cancer 109 786 92 14999791 10.1002/ijc.20041

[b126-bmi-2007-173] Shukla A Ramos-Nino M Mossman B 2003 Cell signaling and transcription factor activation by asbestos in lung injury and disease Int J Biochem Cell Biol 35 1198 209 12757757 10.1016/s1357-2725(02)00315-1

[b127-bmi-2007-173] Sigstad E Dong HP Nielsen S 2005 Quantitative analysis of integrin expression in effusions using flow cytometric immunophenotyping Diagn Cytopathol 33 321 31 10.1002/dc.20282 16240402

[b128-bmi-2007-173] Simsir A Fetsch P Bedrossian CW 2001 Absence of SV-40 large T antigen (Tag) in malignant mesothelioma effusions: an immunocytochemical study Diagn Cytopathol 25 203 7 11599101 10.1002/dc.2039

[b129-bmi-2007-173] Singhal S Wiewrodt R Malden LD 2003 Gene expression profiling of malignant mesothelioma Clin Cancer Res 9 3080 97 12912960

[b130-bmi-2007-173] Sivertsen S Berner A Michael CW 2006 Cadherin expression in ovarian carcinoma and malignant mesothelioma cell effusions Acta Cytol 50 603 7 17152269 10.1159/000326027

[b131-bmi-2007-173] Sivertsen S Hadar R Elloul S 2006 Expression of Snail, Slug and Sip1 in malignant mesothelioma effusions is associated with matrix metalloproteinase, but not with cadherin expression Lung Cancer 54 309 17 16996643 10.1016/j.lungcan.2006.08.010

[b132-bmi-2007-173] Soini Y Kinnula V Kaarteenaho-Wiik R 1999 Apoptosis and expression of apoptosis regulating proteins bcl-2, mcl-1, bcl-X, and bax in malignant mesothelioma Clin Cancer Res 5 3508 15 10589765

[b133-bmi-2007-173] Sterman DH Recio A Vachani A 2005 Long-term follow-up of patients with malignant pleural mesothelioma receiving high-dose adenovirus herpes simplex thymidine kinase/ganciclovir suicide gene therapy Clin Cancer Res 11 7444 53 16243818 10.1158/1078-0432.CCR-05-0405

[b134-bmi-2007-173] Strizzi L Vianale G Catalano A 2001 Basic fibroblast growth factor in mesothelioma pleural effusions: correlation with patient survival and angiogenesis Int J Oncol 18 1093 8 11295061 10.3892/ijo.18.5.1093

[b135-bmi-2007-173] Strizzi L Catalano A Vianale G 2001 Vascular endothelial growth factor is an autocrine growth factor in human malignant mesothelioma J Pathol 193 468 75 11276005 10.1002/path.824

[b136-bmi-2007-173] Sugarbaker PH Welch LS Mohamed F 2003 A review of peritoneal mesothelioma at the Washington Cancer Institute Surg Oncol Clin N Am 12 605 21 14567020 10.1016/s1055-3207(03)00045-0

[b137-bmi-2007-173] Suzuki E Kapoor V Cheung HK 2004 Soluble type II transforming growth factor-beta receptor inhibits established murine malignant mesothelioma tumor growth by augmenting host antitumor immunity Clin Cancer Res 10 5907 18 15355924 10.1158/1078-0432.CCR-03-0611

[b138-bmi-2007-173] Swain WA O’Byrne KJ Faux SP 2004 Activation of p38 MAP kinase by asbestos in rat mesothelial cells is mediated by oxidative stress Am J Physiol Lung Cell Mol Physiol 286 L859 65 14617514 10.1152/ajplung.00162.2003

[b139-bmi-2007-173] Takeichi M 1991 Cadherin cell adhesion receptors as a morphogenetic regulator Science 51 1451 5 2006419 10.1126/science.2006419

[b140-bmi-2007-173] Thirkettle I Harvey P Hasleton PS 2000 Immunoreactivity for cadherins, HGF/SF, met, and erbB-2 in pleural malignant mesotheliomas Histopathology 36 522 8 10849094 10.1046/j.1365-2559.2000.00888.x

[b141-bmi-2007-173] Toyooka S Pass HI Shivapurkar N 2001 Aberrant methylation and simian virus 40 tag sequences in malignant mesothelioma Cancer Res 61 5727 30 11479207

[b142-bmi-2007-173] Urwin D Lake RA 2000 Structure of the Mesothelin/MPF gene and characterization of its promoter Mol Cell Biol Res Commun 3 26 32 10683314 10.1006/mcbr.2000.0181

[b143-bmi-2007-173] van Ruth S Baas P Zoetmulder FAN 2003 Surgical treatment of malignant pleural mesothelioma Chest 123 551 61 12576380 10.1378/chest.123.2.551

[b144-bmi-2007-173] Versnel MA Claesson-Welsh L Hammacher A 1991 Human malignant mesothelioma cell lines express PDGF beta-receptors whereas cultured normal mesothelial cells express predominantly PDGF alpha-receptors Oncogene 6 2005 11 1658707

[b145-bmi-2007-173] Vintman L Nielsen S Berner A 2005 Mitogen-activated protein kinase (MAPK) expression and activation does not differentiate benign from malignant mesothelial cells Cancer 103 2427 33 15830375 10.1002/cncr.21014

[b146-bmi-2007-173] Vogelzang NJ Porta C Mutti L 2005 New agents in the management of advanced mesothelioma Semin Oncol 32 336 50 15988688 10.1053/j.seminoncol.2005.02.010

[b147-bmi-2007-173] Wilson J Balkwill F 2002 The role of cytokines in the epithelial cancer microenvironment Semin Cancer Biol 12 113 20 12027583 10.1006/scbi.2001.0419

[b148-bmi-2007-173] Xia C Xu Z Yuan X 2002 Induction of apoptosis in mesothelioma cells by antisurvivin oligonucleotides Mol Cancer Ther 1 687 94 12479365

[b149-bmi-2007-173] Xio S Li D Vijg J 1995 Codeletion of p15 and p16 in primary malignant mesothelioma Oncogene 11 511 5 7630635

[b150-bmi-2007-173] You L He B Uematsu K 2004 Inhibition of Wnt-1 signaling induces apoptosis in beta-catenin-deficient mesothelioma cells Cancer Res 64 3474 8 15150100 10.1158/0008-5472.CAN-04-0115

[b151-bmi-2007-173] Zanella CL Posada J Tritton TR 1996 Asbestos causes stimulation of the extracellular signal-regulated kinase 1 mitogen-activated protein kinase cascade after phosphorylation of the epidermal growth factor receptor Cancer Res 56 5334 8 8968079

